# Photocatalytic Papers Comprising Au@SnO_2_ Nanocrystals Immobilized on Cellulose Nanofibers for Sustainable Dye Degradation

**DOI:** 10.1021/acsmaterialsau.4c00130

**Published:** 2024-12-13

**Authors:** Yu-Chen Wei, Huai-En Chang, Pulikkutty Subramaniyan, Shan-Chu Yu, Yung-Jung Hsu, Tzu-En Lin

**Affiliations:** † Department of Materials Science and Engineering, National Yang Ming Chiao Tung University, Hsinchu 300093, Taiwan; ‡ Institute of Applied Mechanics, National Taiwan University, Taipei 10617, Taiwan; § Institute of Biomedical Engineering, National Yang Ming Chiao Tung University, Hsinchu 300093, Taiwan; ∥ Center for Emergent Functional Matter Science, National Yang Ming Chiao Tung University, Hsinchu 300093, Taiwan; ⊥ Institute of Integrated Research, Institute of Science Tokyo, Kanagawa 226-8503, Japan

**Keywords:** Au@SnO_2_, core@shell, cellulose, photocatalytic paper, dye degradation

## Abstract

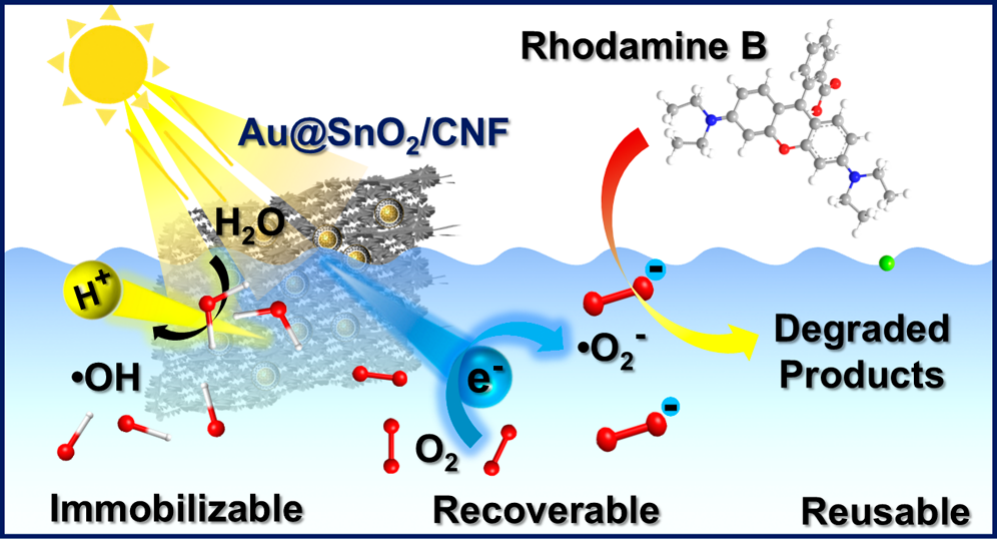

This work presents the synthesis, characterization, and photocatalytic performance of a sophisticated photocatalytic paper comprising Au@SnO_2_ core@shell nanocrystals immobilized on cellulose nanofibers (CNF). The Au@SnO_2_/CNF nanocrystal immobilized paper (NIP) is employed as photocatalysts for degradation of rhodamine B (RhB) under simulated sunlight irradiation. Results reveal that the Au@SnO_2_/CNF NIP exhibits a notable photocatalytic activity driven by efficient charge separation at the interface of Au and SnO_2_. Mechanistic insights into the degradation process indicate that photoexcited electrons in the Au core reduce dissolved oxygen to form superoxide radicals, while photogenerated holes in the SnO_2_ valence band oxidize water to generate hydroxyl radicals. These reactive oxygen species, along with the separated holes themselves, contribute to RhB degradation. Importantly, the Au@SnO_2_/CNF NIP demonstrates remarkable recyclability toward RhB degradation, retaining 88% of its initial activity after 18 degradation cycles, highlighting its potential for sustainable environmental remediation applications.

## 1 Introduction

Industries such as textiles, plastics, and cosmetics generate substantial amounts of wastewater containing various dyes, many of which are toxic, potentially carcinogenic, and pose a significant threat to aquatic ecosystems.^1^ As a result, the removal of these dyes from industrial effluents and the development of advanced water treatment technologies have become critical areas of focus in recent years. Xanthene-based dyes, in particular, have garnered attention due to their carcinogenic and mutagenic effects on humans, their toxicity to aquatic life, and their resistance to conventional biological treatments and natural degradation processes. Rhodamine B (RhB), a widely used xanthene-based dye recognized for its bright pink hue, is notably resistant to standard industrial oxidation methods, contributing to its persistence and potential for environmental contamination.^2^ Prolonged exposure to RhB in the ecosystem poses significant health risks to humans, including increased susceptibility to cancer, organ damage (particularly affecting the liver and kidneys), respiratory and dermal irritation, endocrine disruption, genetic mutations, neurotoxicity, ocular damage, and compromised immune function.^3,4^ Therefore, the removal of RhB from the environment, especially from water systems, remains a critical concern. To effectively treat pollutants such as RhB, a range of technologies have been developed, including photocatalytic degradation,^5−7^ advanced oxidation processes,^8,9^ electrocatalytic degradation,^10,11^ and biodegradation.^12^ Among these, photocatalytic degradation by employing semiconductor photocatalysts has emerged as a particularly promising solution owing to its distinct advantages, including easy experimental setup and environmental friendliness. Till now, a variety of semiconductor photocatalysts have been proposed to realize effective degradation of RhB.

SnO_2_, a typical n-type semiconductor with a direct bandgap, is recognized for its chemical stability and high electron mobility.^13^ However, its photocatalytic efficiency is limited by the rapid recombination of photogenerated charge carriers in its single-component form.^14^ To mitigate this limitation, various band structure engineering strategies have been developed, including metal nanoparticle deposition,^15^ the formation of type-II heterostructures,^16^ and the design of *Z*-scheme^17−20^ and *S*-scheme heterostructures.^21,22^ Among these, metal nanoparticle deposition has been particularly effective in enhancing charge carrier transfer and reducing recombination.^23−25^ The Fermi levels (*E*_F_) of metals are generally less cathodic than the conduction band edge of semiconductors, promoting the preferential transfer of photoexcited electrons from the semiconductor to the metal while retaining holes in the semiconductor. This mechanism significantly enhances charge separation and, consequently, photocatalytic activity.^26,27^ Additionally, metal nanoparticles such as Au, Ag, and Cu exhibit localized surface plasmon resonance (LSPR), which boosts photocatalytic performance by enabling additional charge carrier generation through processes such as hot electron injection,^28^ resonance energy transfer,^29^ and electromagnetic field amplification.^30^ By exploiting the plasmonic properties of metal nanoparticles, semiconductor photocatalysts can be tailored to achieve greater efficiency in a wide range of applications.^31,32^

Incorporating Au nanoparticles into a core@shell architecture with SnO_2_ is a highly effective strategy for enhancing the photocatalytic activity of SnO_2_. Compared to other heterostructures, core@shell nanocrystals offer significant advantages, such as enhanced charge separation and reduced charge recombination due to the intimate interface between the core and shell, which enables more efficient charge carrier transfer. Moreover, the core@shell structure provides increased stability by protecting the core from corrosion and detachment, resulting in greater durability in photocatalytic applications. The combination of Au and SnO_2_ in this configuration is thus expected to deliver superior photocatalytic performance, particularly in the degradation of organic pollutants, such as RhB. Although core@shell nanocrystals demonstrate promising photocatalytic performance, their recovery and reuse in powder form present substantial challenges.^33,34^ Powder-like substances are prone to dispersing into solution and are often difficult to recycle effectively.^35^ To address this issue, the integration of core@shell nanocrystals with cellulose nanofibers (CNF) in a paper-based architecture has been proposed. Cellulose, a naturally occurring biodegradable biopolymer found in plants and bacteria,^36^ provides an excellent platform for immobilizing photocatalyst powders, enabling sustainable photocatalytic operations. Upon oxidation treatment, CNF is rich in carboxyl groups, facilitating strong hydrogen bonding interactions and allowing for further chemical modification.^37^ These carboxyl groups can form tight bonds with metal oxides through esterification, improving recovery and reuse potential.^38^ The robust bonding between metal oxides and CNF allows for easy recovery, reusability, and the development of environmentally friendly catalysts. In this study, a paper-structured photocatalyst was fabricated by immobilizing Au@SnO_2_ core@shell nanocrystals on bamboo wastepaper derived CNF (Au@SnO_2_/CNF). The photocatalytic properties of the Au@SnO_2_/CNF nanocrystal immobilized paper (NIP) were investigated for the degradation of RhB. Results show that the fabricated photocatalytic paper demonstrated sustainable performance, highlighting its potential for various photocatalytic applications.

## 2 Experimental Section

### 2.1 Materials and Chemicals

The purchase of gold chloride trihydrate (HAuCl_4_·3H_2_O, 99.9%), sodium citrate dihydrate (C_6_H_5_Na_3_O_7_·2H_2_O, 99.0%), RhB (97.0%), and sodium stannate trihydrate (Na_2_Sn_2_O_3_·3H_2_O, 95%), potassium cyanide (KCN, 96.0%), silver nitrate (AgNO_3_, 99.9%), ethylenediaminetetraacetic acid (EDTA, 99.0%), isopropanol (IPA, 99.9%), and benzoquinone (BQ, 98.0%) was made from Sigma-Aldrich. Sodium hydroxide (NaOH, 98.5%) and sodium chlorite (NaClO_2_, 80%) were acquired from Showa chemicals. 2,2,6,6 Tetramethylpiperidinyloxy (TEMPO, (CH_2_)_3_(CMe_2_)_2_NO, 98%) and sodium hypochlorite solution (NaClO, 4%) were purchased from Thermo Scientific Corporation. The acetic acid (CH_3_COOH, 99.7%) was purchased from Tokyo Chemical Industry.

### 2.2 Fabrication of Photocatalytic Paper

#### 2.2.1 Synthesis of Au@SnO_2_

**Scheme 1:**
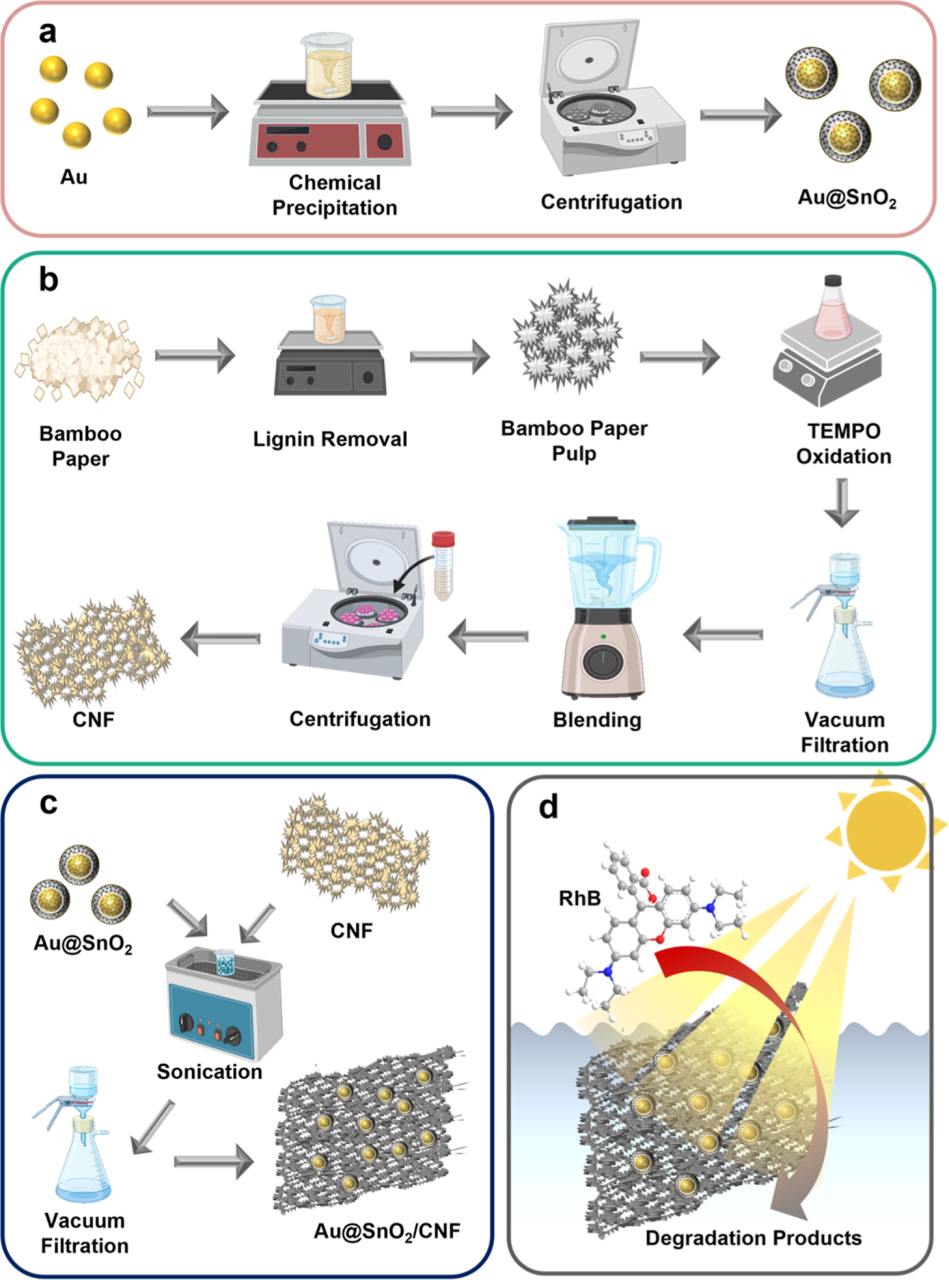
Schematic Illustration of Fabricating Photocatalytic Papers for RhB Degradation The processes involve (a) the synthesis of Au@SnO_2_, (b) the synthesis of CNF, (c) the immobilization of Au@SnO_2_ on CNF, (d) the use of Au@SnO_2_/CNF NIP for RhB degradation.

As depicted in Scheme 1a, a suspension of Au nanoparticles with an average size of 15 nm was synthesized using the citrate reduction method as described in a previous publication.^39^ The Au nanoparticles were then coated with a SnO_2_ layer through a chemical precipitation method.^40^ To synthesize Au@SnO_2_ core@shell nanocrystals, the Au nanoparticle solution was first adjusted to a pH of 13. Na_2_SnO_4_ was added, and the mixture was stirred at 90 °C for 10 min. The reaction mixture was subsequently subjected to hydrothermal treatment at 120 °C for 1 h. After completion, the resulting solution was centrifuged and rinsed three times with deionized (DI) water to obtain the Au@SnO_2_ core@shell nanocrystals. For comparison purpose, pure SnO_2_ was also synthesized by etching the Au core of Au@SnO_2_ using 0.1 M KCN.

#### 2.2.2 Synthesis of CNF

As illustrated in Scheme 1b, 10 g of bamboo raw edge paper was blended with DI water to create a 2.0% solution (500 mL). To this, 1.25 mol of NaOH was added, and the mixture was stirred at 80 °C and 650 rpm for 2 h to prepare the pulp. The pulp was then separated using vacuum filtration and washed several times with DI water to remove any residual NaOH. To delignify the treated pulp, it was dispersed in 160 mL of DI water, followed by the addition of 1.5 g of 80% NaClO_2_ and 200 μL of CH_3_COOH. The mixture was then heated to 80 °C and maintained for 1 h. For TEMPO-mediated oxidation,^41,42^ 5 g of the pretreated pulp was mixed with 500 mL of 0.05 M phosphate buffer solution (pH 7). Subsequently, 200 mg of TEMPO, 4 g of NaClO_2_, and 20 mL of 4.0% NaClO_2_ solution were added. The container was sealed and heated to 60 °C for 18 h. The resulting mixture was filtered under vacuum, washed three times with DI water, and a 2.5 wt % fiber solution was prepared. The fiber solution was blended for 30 min, pausing every 5 min to prevent overheating, then centrifuged at 4000 rpm for 30 min. The supernatant was then concentrated by heating until the solution reached 2.5 wt %.

#### 2.2.3 Fabrication of Au@SnO_2_/CNF

As shown in Scheme 1c, to achieve complete dispersion, 10 mg of Au@SnO_2_ and 20 mg of CNF were added to 40 mL of DI water and subjected to vigorous ultrasonication for 30 min. The resulting Au@SnO_2_/CNF suspension was filtered using suction filtration through a Whatman membrane filter (47 mm diameter, 0.1 μm pore size). The Au@SnO_2_/CNF NIP with a diameter of 3 cm was easily recovered from the filter by drying it for 30 min at 45 °C. As depicted in Scheme 1d, the fabricated Au@SnO_2_/CNF NIP was then used for RhB degradation under simulated sunlight irradiation to evaluate its photocatalytic performance. For comparison purpose, pure SnO_2_ (9.2 mg) was also immobilized on CNF (20 mg) to form SnO_2_/CNF NIP using the same procedures. Note that the loaded SnO_2_ amount was adjusted to equate the SnO_2_ content (9.2 mg) of 10 mg Au@Sn_2_O.

### 2.3 RhB Degradation

The photocatalytic performance of the as-fabricated Au@SnO_2_/CNF NIP was evaluated through the degradation of RhB. A given amount of Au@SnO_2_/CNF NIP (30 mg, composed of 10 mg Au@SnO_2_ and 20 mg CNF) was immersed in 20 mL of an aqueous RhB solution with a concentration of 10^–6^ M and exposed to simulated solar light with an intensity of 100 mW/cm^2^. Samples were collected at 20 min intervals, and the concentration of RhB was measured using a UV–vis spectrophotometer at a wavelength of 554 nm. The photocatalytic performance of pristine CNF (20 mg), individual Au@SnO_2_ (10 mg), pure SnO_2_ (9.2 mg), and SnO_2_/CNF (29.2 mg, composed of 9.2 mg pure SnO_2_ and 20 mg CNF) was also examined and compared. The adsorption experiments were conducted on two representative samples: individual Au@SnO_2_ and Au@SnO_2_/CNF NIP. These experiments were conducted by dispersing the samples in RhB solution (20 mL, 10^–6^ M) under dark conditions with continuous stirring for 20 min to evaluate adsorption. Subsequently, light irradiation was applied to initiate and monitor the photocatalytic degradation of RhB. For the recycling tests, each cycle of RhB degradation was conducted for 120 min of illumination. In the experiments with Au@SnO_2_/CNF, the photocatalytic paper was removed from the used solution at the end of each cycle, rinsed with DI water, and placed in a fresh RhB solution to begin the subsequent cycle. For the experiments using individual Au@SnO_2_, the photocatalyst powders were recovered by centrifugation and reused in the next cycle of RhB degradation.

### 2.4 Scavenger Experiments

To identify the primary reactive species, four distinct radical scavengers were employed: AgNO_3_, EDTA, IPA, and BQ.^43^ These scavengers were specifically selected to target electrons, holes, ^•^OH, and ^•^O_2_^–^ radicals, respectively, generated by Au@SnO_2_/CNF during the photocatalytic degradation of RhB. In the typical experimental procedure, 1 mM of each scavenger was added to the photocatalytic reaction solution for RhB degradation. Changes in RhB concentration were carefully monitored and analyzed in comparison with a control experiment conducted without any radical scavengers.

### 2.5 Characterizations

The morphology of the samples was characterized using scanning electron microscopy (SEM, Hitachi SU-8010) operated at 15 kV and transmission electron microscopy (TEM, JEOL JEM-F200) operated at 200 kV. X-ray diffraction (XRD) patterns were recorded using a Bruker D2 Phaser with Cu Kα radiation (λ = 1.5406 Å at 30 kV and 10 mA) to analyze the sample composition and crystallinity. Fourier-transform infrared (FTIR) spectroscopy (PerkinElmer Spectrum One) was used to identify surface functional groups. UV–vis absorption spectra of the solutions were obtained with a Hitachi U3900H spectrophotometer to study the optical properties. An integrating sphere is attached to the spectrophotometer, enabling the collection of diffuse reflectance spectra (DRS) on powder samples and CNF papers. Chemical state analysis was performed via X-ray photoelectron spectroscopy (XPS, Thermo Fisher Scientific ESCALAB Xi+) with Al Kα radiation. Ultraviolet photoelectron spectroscopy (UPS) spectra were acquired using a Thermo Fisher Scientific ESCALAB Xi+ with He I (*h*υ = 21.22 eV) as the excitation source. A xenon lamp equipped with an AM 1.5 G filter in a solar simulator (Newport, LCS-100, 94011A) provided an irradiance of 100 mW/cm^2^, serving as the irradiation source for the degradation of RhB. Brunauer–Emmett–Teller (BET) surface areas of the samples (100 mg) were determined from the N_2_ adsorption–desorption isotherms (Micromeritics ASAP2020). Steady-state photoluminescence (PL) spectra were recorded (Hitachi, F-4500) to explore interfacial charge transfer of the samples. The excitation wavelength was set at 325 nm.

## 3 Results and Discussion

### 3.1 Microstructures, Optical Properties and Chemical States

**Figure 1:**
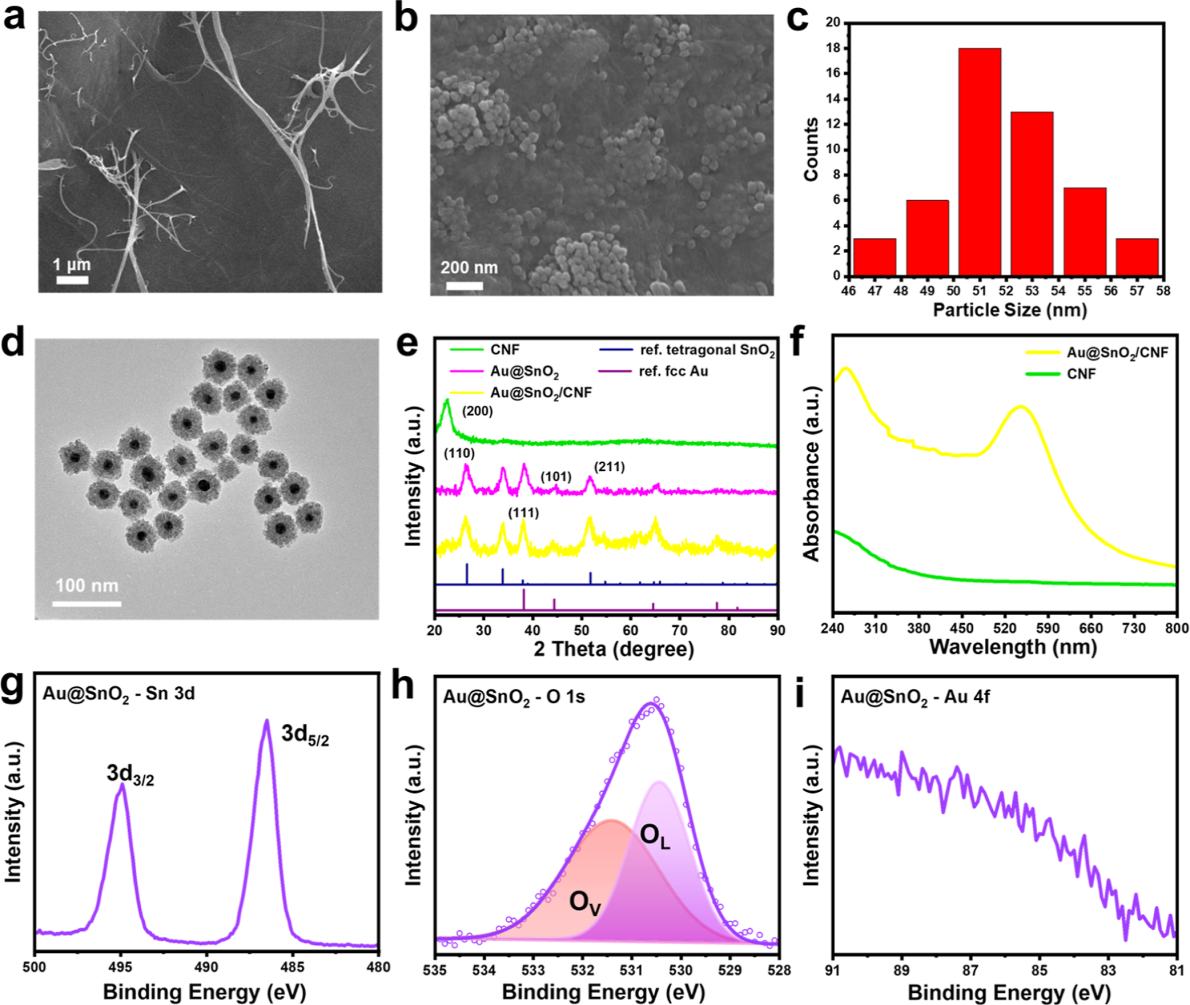
SEM images of (a) pristine CNF, (b) Au@SnO_2_/CNF NIP. (c) Particle size distribution, (d) TEM image of individual Au@SnO_2_. (e) XRD patterns of relevant samples with the reference patterns of fcc Au (JCPDS #04-0784) and tetragonal SnO_2_ (JCPDS #41-1445) included. (f) UV–vis DRS spectra of pristine CNF and Au@SnO_2_/CNF NIP. XPS spectra of (g) Sn 3d, (h) O 1s, (i) Au 4f core levels for individual Au@SnO_2_.

SEM was conducted to examine the microstructural features of CNF and Au@SnO_2_. In Figure 1a, the SEM image of pristine CNF reveals a fibrous structure with nanometer-scale diameter, showcasing the typical morphology of carbon nanofibers. Additional SEM images of pristine CNF at various magnifications were displayed in Figure S1 to illustrate its microstructural characteristics. The CNF exhibits intact, interlaced fibrous nanostructures with protruding nanofibers on its surface. The diameters of these nanofibers range from 50 to 250 nm. In Figure S2, the as-synthesized Au@SnO_2_ nanocrystals exhibit a particle-shaped morphology with an average primary size of 52.1 nm. Figure 1b shows the SEM image of CNF after the introduction of Au@SnO_2_ nanocrystals, where the CNF surface is fully coated with densely packed nanocrystals, indicating successful deposition of Au@SnO_2_ and strong adhesion between CNF and Au@SnO_2_. Note that upon TEMPO oxidation, CNF was enriched in carboxyl moieties that can form hydrogen bonds with the surface hydroxyl groups of Au@SnO_2_, providing a substantial binding force between CNF and Au@SnO_2_. The photographs of pristine CNF, pure SnO_2_, SnO_2_/CNF, individual Au@SnO_2_, and Au@SnO_2_/CNF were taken to highlight their bulk appearances. As shown in Figure S3, pristine CNF appears as a pale-colored circular paper. Upon immobilization of SnO_2_ and Au@SnO_2_, the paper transitions to white and dark brown, respectively.

The FTIR spectra for pristine CNF and Au@SnO_2_/CNF were also studied. The spectra, presented in Figure S4, reveal that pristine CNF and Au@SnO_2_/CNF exhibit comparable absorption features characteristic of the functional groups of cellulose. Specifically, the stretching and bending vibrations of the C–H bonds are observed at 2898 and 894 cm^–1^, respectively.^44^ A prominent absorption at 1014 cm^–1^ and a small band at 1421 cm^–1^ are attributed to C–O and H–C–H functional groups, respectively.^45^ Additionally, distinctive absorptions at 1602 and 3294 cm^–1^ correspond to the bending and stretching vibrations of OH groups.^46^ These spectral similarities confirm the incorporation of CNF in the Au@SnO_2_/CNF. The particle size distribution of the Au@SnO_2_ nanocrystals, as depicted in Figure 1c, exhibits a relatively narrow spread, with the average particle size centered around 51.8 ± 2.4 nm. This size distribution confirms the consistency in nanocrystal introduction and highlights the uniformity of the deposition process. Figure 1d presents a TEM image of individual Au@SnO_2_ nanocrystals, highlighting the high microstructural integrity of the core@shell configuration. The Au core exhibits an average size of 16.3 ± 2.5 nm, while the SnO_2_ shell has a uniform thickness of 18.0 ± 2.0 nm. XRD analysis was utilized to assess the crystallinity and phase composition of pristine CNF, individual Au@SnO_2_, and Au@SnO_2_/CNF NIP. As depicted in Figure 1e, the sharp diffraction peak at a 2θ angle of 22° corresponds to the (200) plane of pristine CNF, indicative of the hexagonal crystalline structure of sp^2^ carbon.^47^ For pristine Au@SnO_2_, the weak but discernible peaks at 38.26 and 44.6° correspond to the face-centered cubic (fcc) phase of metallic Au, while the dominant SnO_2_ peaks at 26.48, 33.74, and 51.5° are attributed to the (110), (101), and (211) planes of tetragonal SnO_2_, respectively. The XRD pattern of theAu@SnO_2_/CNF NIP shows identical fcc Au and tetragonal SnO_2_ peaks as those of Au@SnO_2_, with the CNF peak being absent due to the signal interference by Au@SnO_2_. The current Au@SnO_2_/CNF is composed of a Au@SnO_2_ to CNF ratio of 1:2. While X-rays can penetrate the entire Au@SnO_2_/CNF, the diffraction peak of CNF may be obscured by the strong scattering signals from the abundant Au@SnO_2_. To validate this argument, additional XRD measurements on Au@SnO_2_/CNF synthesized with a lower loading of Au@SnO_2_ were conducted. As shown in Figure S5, an observable (200) peak of CNF appears when the Au@SnO_2_ to CNF ratio is adjusted to 1:5. This result confirms that the absence of the (200) peak for CNF in the original Au@SnO_2_/CNF was due to signal interference from the strong scattering of Au@SnO_2_.

UV–vis absorption spectroscopy was employed to investigate the optical properties of the Au@SnO_2_/CNF NIP. As shown in Figure 1f, pristine CNF exhibits a strong π–π* transition, with absorption extending into the mid-UV region.^48^ The Au@SnO_2_/CNF NIP displays two distinct absorbance bands at 269 and 530 nm, corresponding to contributions from the CNF, SnO_2_, and Au components. The absorption band at 269 nm is at-tributed to the combined bandgap absorption of SnO_2_ and the π–π* transition of CNF, while the band at 530 nm is associated with the LSPR of Au. The slight red shift of the LSPR band from 520 nm, typically observed in Au colloids, to 530 nm in Au@SnO_2_/CNF NIP is attributed to the dielectric influence of the SnO_2_ matrix.^49^ XPS was utilized to investigate the chemical states for Au@SnO_2_. As shown in Figure 1g, the XPS spectra of the Sn 3d region for individual Au@SnO_2_ display pronounced peaks at 486.4 and 494.8 eV, corresponding to the Sn 3d_5/2_ and Sn 3d_3/2_ orbitals, respectively, confirming the presence of Sn^4+^ oxidation state.^50^ In Figure 1h, the O 1s spectrum reveals two deconvoluted peaks, attributed to lattice oxygen (O_L_) at 530.3 eV and oxygen vacancies (O_V_) at 531.4 eV.^51^ The Au 4f doublets are notably absent in Figure 1i, which can be attributed to the thick SnO_2_ shell (around 18 nm) encapsulating the Au core, preventing photoelectrons from escaping.

### 3.2 Photocatalytic Degradation of RhB

**Figure 2:**
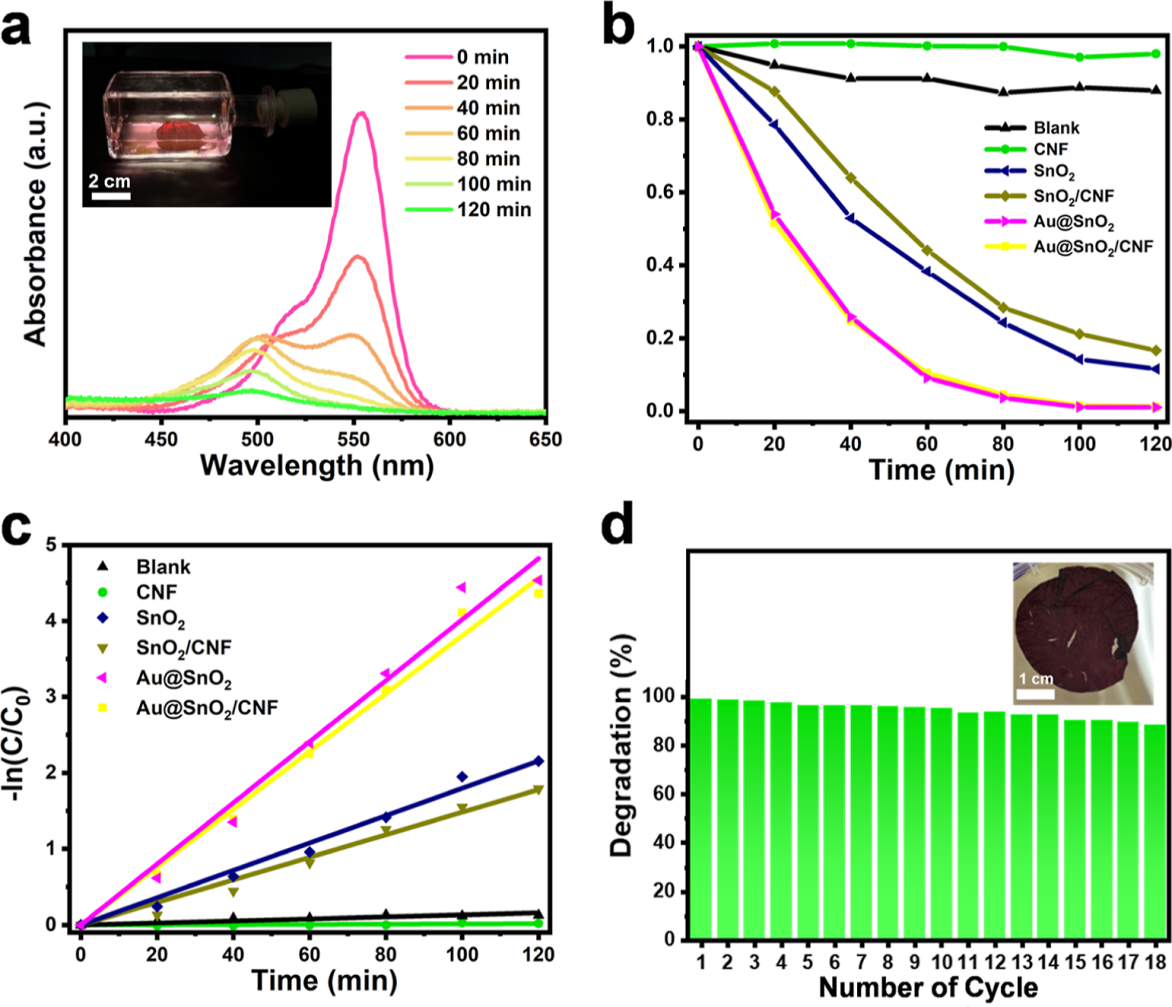
(a) UV–vis absorption spectra of RhB under different irradiation times in the presence of Au@SnO_2_/CNF. Inset shows the experimental setup. (b) *C*/*C*_0_ versus irradiation time plots for RhB degradation without any photocatalyst (blank) and in the presence of pristine CNF, pure SnO_2_, SnO_2_/CNF, individual Au@SnO_2_, and Au@SnO_2_/CNF. (c) −ln(*C*/*C*_0_) versus irradiation time plots with the fitting results included. (d) Degradation percentage of RhB in the presence of Au@SnO_2_/CNF NIP over multiple cycles. Inset shows the photo of the used Au@SnO_2_/CNF NIP.

The photocatalytic degradation of RhB was carried out using Au@SnO_2_/CNF NIP under simulated sunlight irradiation. The experimental setup, shown in the inset of Figure 2a, involves bottom-up light irradiation onto the reactor containing the RhB solution and Au@SnO_2_/CNF NIP. The progressive decrease in the characteristic RhB absorbance at 554 nm indicates effective degradation. Additionally, a noticeable blue shift in the absorption maximum is observed after 40 min of irradiation. It is well-established that the photocatalytic degradation of RhB proceeds through two competitive processes.^52−54^ One involves the destruction of the dye chromogen, characterized by a loss of absorbance at 554 nm. The other is an *N*-demethylation reaction, which generates a series of *N*-demethylated intermediates, leading to a blue shift in the absorption maximum from 554 to 498 nm. This spectral evolution provides clear evidence for the destructive degradation of RhB during the photocatalytic process. In this study, RhB concentration was quantified based on the absorbance peak at 554 nm. To elucidate the reaction kinetics, the normalized concentration (*C*/*C*_0_) versus irradiation time was analyzed.

As presented in Figure 2b, in the absence of a photocatalyst, RhB exhibited fairly slight degradation over 120 min of irradiation, demonstrating its high resistance under sunlight. However, in the presence of Au@SnO_2_/CNF NIP, RhB was fully degraded within 120 min of irradiation. The photocatalytic performance of pristine CNF, individual Au@SnO_2_, pure SnO_2_ and SnO_2_/CNF as additional reference samples was also evaluated for comparison. Several significant observations can be drawn from the findings. First, pristine CNF exhibited negligible activity in RhB degradation, emphasizing its role as an inert support for immobilizing photocatalysts. To examine the possible electronic interaction between CNF and Au@SnO_2_, XPS Sn 3d spectra of individual Au@SnO_2_ and Au@SnO_2_/CNF were compared. As shown in Figure S6, the Sn 3d doublet peaks exhibit no significant differences in binding energy, indicating the absence of electronic interaction between Au@SnO_2_ and CNF in the Au@SnO_2_/CNF. This result supports our conclusion that CNF functions as an inert support for immobilizing photocatalysts. Second, Au@SnO_2_ demonstrated superior photocatalytic performance compared to pure SnO_2_ whether in its individual form or immobilized on CNF. This enhanced activity can be attributed to improved charge separation facilitated by interfacial charge transfer between Au and SnO_2_. To examine the improved charge separation for Au@SnO_2_, steady-state PL spectra of individual Au@SnO_2_ and pure SnO_2_ were compared.^55,56^ As shown in Figure S7, Au@SnO_2_ exhibits significantly lower PL intensity compared to pure SnO_2_. This reduction is attributed to the preferential transfer of photoexcited electrons from SnO_2_ to Au, which effectively suppresses electron–hole recombination of SnO_2_ component in Au@SnO_2_. The substantial decrease in PL intensity provides strong evidence for the enhanced charge separation in Au@SnO_2_. Third, the photocatalytic efficiency of both Au@SnO_2_ and pure SnO_2_ slightly diminished upon immobilization on CNF. This reduction is likely due to a decrease in specific surface area available for catalytic reactions when the nanocrystals are immobilized on the CNF. The BET measurements were further conducted to evaluate the specific surface areas of the samples. The results show that pristine CNF, individual Au@SnO_2_, and Au@SnO_2_/CNF possess specific surface areas of 0.9, 72.3, and 29.8 m^2^/g, respectively. The reduced surface area of Au@SnO_2_/CNF, compared to individual Au@SnO_2_, can be recognized from the microstructural features observed in the corresponding SEM image. As shown in Figure 1b, the Au@SnO_2_ nanocrystals immobilized on the CNF surface exhibit partial aggregation, which likely contributes to the reduction in specific surface area. This microstructural characteristic also influences the adsorption capacity for RhB molecules and the resulting photocatalytic performance. As shown in Figure S8, individual Au@SnO_2_ adsorbed 13.2% of RhB within 20 min in the dark, whereas Au@SnO_2_/CNF adsorbed 7.0% under the same conditions. The slightly lower adsorption capacity of Au@SnO_2_/CNF correlates with its diminished photocatalytic performance. The degradation kinetics were modeled using the Langmuir–Hinshelwood equation, and the apparent rate constant (*k*_RhB_) was derived from the linear fit of −ln(*C*/*C*_0_) versus time (*t*), following the expression −ln(*C*/*C*_0_) = *k*_RhB_ × *t*.^57^ As shown in Figure 2c, the exponential relationship confirms the validity of the model, with a k_RhB_ value of 1.33 × 10^–4^, 1.79 × 10^–2^, 1.48 × 10^–2^, 4.02 × 10^–2^, and 3.80 × 10^–2^ min^–1^ for pristine CNF, pure SnO_2_, SnO_2_/CNF, individual Au@SnO_2_, and Au@SnO_2_/CNF, respectively.

It should be spotlighted that the paper-like structure of Au@SnO_2_/CNF NIP can allow for easy recovery after RhB degradation. By virtue of this attribute, the photocatalytic activity of Au@SnO_2_/CNF NIP can be retained across multiple cycles. As demonstrated in Figure 2d, Au@SnO_2_/CNF NIP maintained 88% of its initial activity even after 18 cycles of RhB degradation. The minor loss in activity is attributed to the slight detachment of Au@SnO_2_ from the CNF, likely caused by the mechanical movement of the Au@SnO_2_/CNF NIP during each cycle. The intact appearance of the used Au@SnO_2_/CNF NIP can be further highlighted by the inset photo. It should be noted that the primary function of CNF is to facilitate the recovery and reusability of the photocatalyst powders. To emphasize this aspect, stability tests on individual Au@SnO_2_ for RhB degradation were conducted and the sustainable performance was compared with that of Au@SnO_2_/CNF NIP. As shown in Figure S9, the results indicate that after nine cycles, the degradation efficiency of individual Au@SnO_2_ decreases to approximately 80%, which is notably lower than that of Au@SnO_2_/CNF (around 95%). This decline is attributed to material loss during the recovery process for the nonimmobilized Au@SnO_2_, underscoring the advantage of incorporating CNF in preserving photocatalytic performance over multiple cycles. Table 1 provides a performance comparison of the state-of-the-art cellulose-based photocatalytic papers developed in the past three years for dye degradation. In terms of cycling number and retained degradation percentage, the Au@SnO_2_/CNF NIP demonstrates superior performance to most of the previously reported systems, underscoring its exceptional photocatalytic capacity for environmental remediation applications.

**Table 1:** Performance Comparison of the State-of-the-Art Cellulose-Based Photocatalytic Papers Developed for Dye Degradation

photocatalytic paper	light source	dye molecule	cycling number	retained degradation percentage	references
Au@SnO_2_/CNF	AM 1.5 G	RhB	18	88%	this work
aCC/TiO_2_	UV, λ = 254 nm	methyl orange	10	95%	(58)
bCu_2_O/Ag_2_MoO_4_/CF	visible, λ > 420 nm	reactive blue 19	5	86%	(59)
cellulose-TiO_2_	visible	RhB	3	65, 75, 90%	(60)
cellulose hydrogel/TiO_2_	UV	methylene blue	5	80%	(61)
Cu_2_O–ZnO/cellulose	UV–visible	methyl orange	5	83%	(62)

a CC stands for cotton cellulose.

b CF stands for cellulose fiber.

**Figure 3:**
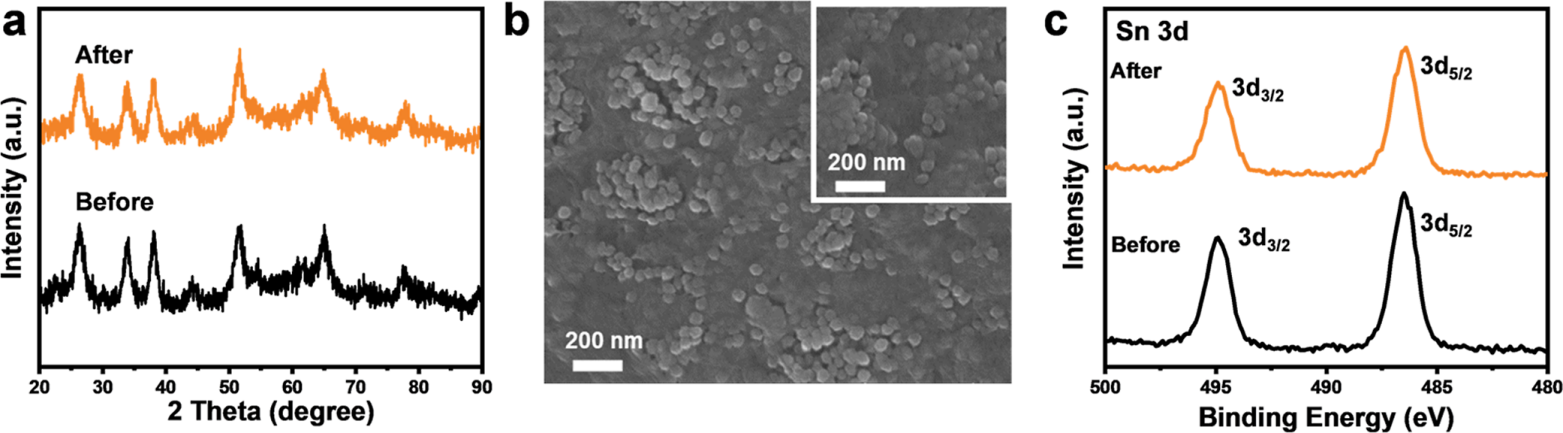
(a) XRD patterns, (b) SEM images, (c) Sn 3d XPS spectra of Au@SnO_2_/CNF NIP before and after multiple cycles of RhB degradation. Inset in (b) shows the image of the used sample.

Following multiple cycles of RhB degradation, the used Au@SnO_2_/CNF NIP was subjected to XRD, XPS, and SEM analyses to assess its structural integrity and chemical stability. Figure 3a presents the XRD patterns of Au@SnO_2_/CNF NIP before and after repeated degradation cycles, revealing no significant changes, thereby confirming its crystalline stability. The SEM image in Figure 3b of the used Au@SnO_2_/CNF NIP closely resembles the as-fabricated one, further demonstrating its excellent structural integrity. XPS spectra of the Sn 3d region, shown in Figure 3c, illustrate that no noticeable alterations occurred in the chemical state of Au@SnO_2_/CNF NIP after RhB degradation. To assess the robustness of Au@SnO_2_/CNF NIP, a folding test was performed on Au@SnO_2_/CNF by folding the paper into a quarter-circle and then unfolding it, both before and after used in recycling tests of RhB degradation. As shown in Figure S10, the Au@SnO_2_/CNF NIP exhibited no visible damage, indicating good mechanical stability and flexibility under these conditions. Furthermore, the apparent color of the Au@SnO_2_/CNF NIP did not change significantly after being used in the recycling tests. Additionally, FTIR measurements were performed on the Au@SnO_2_/CNF NIP both before and after repeated use. As illustrated in Figure S11, no additional peaks were observed, confirming that no residual RhB remained on the NIP after the long-term photocatalytic reaction. These findings highlight the remarkable structural robustness and chemical resilience of the Au@SnO_2_/CNF NIP, reinforcing its potential as a reusable photocatalytic paper for long-term sustainable photocatalytic applications.

### 3.3 Photocatalytic Mechanism

**Figure 4:**
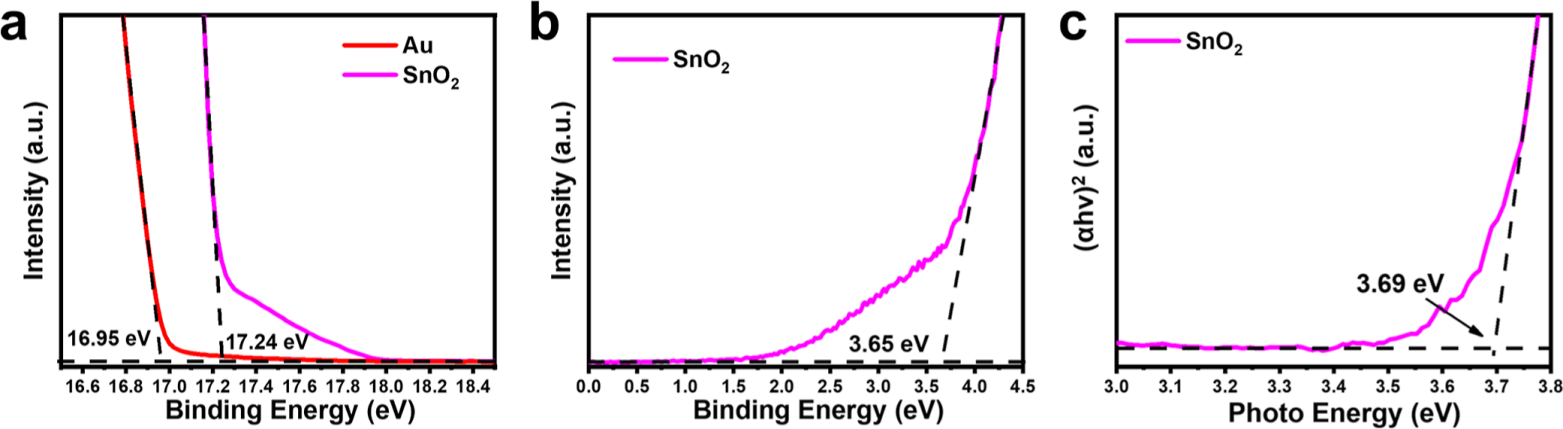
UPS spectra for pure Au and pure SnO_2_ recorded at (a) secondary-electron cutoff region and (b) enlarged valence band region. (c) Tauc plots derived from DRS spectrum for pure SnO_2_.

To provide a more comprehensive understanding of the photocatalytic mechanism, RhB degradation experiments in the presence of radical scavengers were conducted. Specifically, AgNO_3_, EDTA, IPA, and BQ were added to the photocatalytic solution to selectively trap electrons, holes, ^•^OH, and ^•^O_2_^–^, respectively. A substantial decrease in photocatalytic efficiency upon the addition of a specific scavenger indicates the predominant role of the corresponding reactive species in the degradation process. As shown in Figure S12, the addition of AgNO_3_ and BQ significantly suppressed the photocatalytic activity of Au@SnO_2_/CNF, underscoring the crucial roles of electrons and ^•^O_2_^–^ in RhB degradation. In contrast, the presence of IPA and EDTA caused a moderate reduction in photocatalytic performance, suggesting that ^•^OH and holes contribute partially to the degradation process. Based on these findings, we propose the following photocatalytic mechanism. Upon light irradiation, photoexcited electrons from the conduction band of SnO_2_ are preferentially transferred to the E_F_ of Au, where they react with dissolved oxygen to generate ^•^O_2_^–^ as the primary reactive species. Meanwhile, the accumulated holes in the valence band of SnO_2_ oxidize water to produce ^•^OH, which also participate in the RhB degradation. To better interpret the photocatalytic mechanism, the band structure of Au@SnO_2_ was constructed by performing UPS measurements on the two constituent components, including pure Au nanoparticles and pure SnO_2_. As shown in Figure 4a, the high-energy region of the spectra displays a sharp cutoff edge. From the intersection of the tangent with the energy axis, the cutoff energies were determined to be 16.95 eV for pure Au and 17.24 eV for pure SnO_2_. The difference between the incident photon energy (21.22 eV) and these cutoff energies corresponds to the work function of the samples,^63^ calculated as 4.27 eV for Au and 3.98 eV for SnO_2_. Figure 4b shows the spectra near the *E*_F_ level, providing information about the valence band edge (*E*_VB_) of the samples. Using linear extrapolation, the *E*_VB_–*E*_F_ value for pure SnO_2_ was found to be 3.65 eV. By adding the work function, the *E*_VB_ position relative to the vacuum level was determined to be −7.63 eV for pure SnO_2_. Adding the optical bandgap value from Figure 4c, the conduction band edge (*E*_CB_) relative to the vacuum level was estimated to be −3.94 eV for pure SnO_2_. The proximity of the *E*_F_ level to the *E*_CB_ reflects the n-type semiconductor characteristics of pure SnO_2_,^64,65^ possibly attributed to the presence of intrinsic oxygen vacancies.

**Figure 5:**
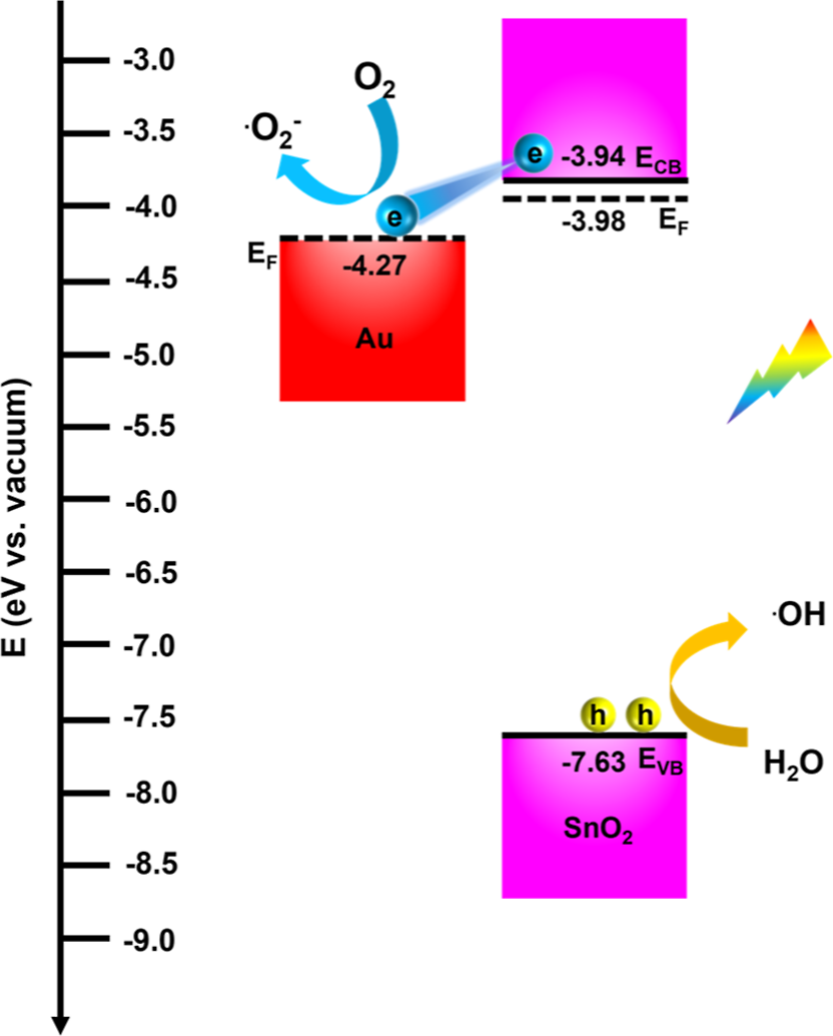
Proposed mechanism of RhB photocatalytic degradation in the presence of Au@SnO_2_/CNF NIP.

Using these energy levels, a plausible band structure is proposed in Figure 5 to elucidate the charge transfer scenario in Au@SnO_2_. For the Au@SnO_2_ system, photoexcited electrons from the conduction band of SnO_2_ preferentially transfer to the *E*_F_ of Au due to the more cathodic *E*_CB_ of SnO_2_ (−3.94 eV vs vacuum) compared to the *E*_F_ of Au (−4.27 eV vs vacuum). This electron transfer leaves the photogenerated holes in the valence band of SnO_2_, resulting in spatial separation of charge carriers, which is advantageous for photocatalytic degradation. The photocatalytic mechanism of RhB degradation over Au@SnO_2_/CNF NIP is further interpreted based on this band structure. The comparative results reveal that CNF serves as an inert support for immobilizing photocatalysts, while Au acts as a charge separation enhancer, facilitating efficient electron transfer at the Au/SnO_2_ interface. Furthermore, due to the large bandgap of SnO_2_ (3.69 eV, as estimated from Figure 4c), the photocatalytic degradation of RhB is primarily driven by UV light. The accumulated photoexcited electrons at Au have a reducing power greater than the reduction potential of O_2_/^•^O_2_^–^ (−4.45 eV vs vacuum),^66^ enabling these electrons to reduce dissolved oxygen molecules to superoxide radicals (^•^O_2_^–^), which play a crucial role in RhB degradation. Simultaneously, the photogenerated holes remaining in the valence band of SnO_2_ exhibit strong oxidizing power, directly facilitating the degradation of RhB. Moreover, these reactive holes, with an oxidizing power higher than the oxidation potentials of H_2_O/^•^OH (−7.18 eV vs vacuum),^67^ can also react with water to generate hydroxyl radicals (^•^OH), further contributing to the degradation of RhB.^68^

## 4 Conclusions

In conclusion, this work introduces an innovative photocatalytic paper comprising Au@SnO_2_ core@shell nanocrystals immobilized on CNF, marking a significant advancement in sustainable dye degradation technologies. The unique core@shell architecture of Au@SnO_2_ enables efficient charge separation at the Au/SnO_2_ interface, which drives the generation of reactive oxygen species critical for RhB degradation. A notable highlight is the remarkable recyclability of the photocatalytic paper, retaining 88% of its activity after 18 cycles, demonstrating both robustness and sustainability. The structural integrity and chemical stability of the photocatalytic paper, as validated by XRD, SEM, XPS, FTIR analyses and folding tests, ensure its practical applicability. This work establishes the Au@SnO_2_/CNF NIP as a highly durable and efficient photocatalyst, paving the way for scalable environmental remediation solutions.

## Supporting Information Available

SEM images, FTIR spectra, XRD patterns, XPS spectra, PL spectra of the samples; photographs of CNF, SnO_2_ and Au@SnO_2_; results of RhB adsorption, scavenger experiments and recycling tests (PDF)
