# Supplementary material for: Photocatalytic Papers Comprising Au@SnO2 Nanocrystals Immobilized on Cellulose Nanofibers for Sustainable Dye Degradation
Source: ACS Mater Au. 2024 Dec 13;5(2):320–30. doi: 10.1021/acsmaterialsau.4c00130 (PMC11907298; doi:10.1021/acsmaterialsau.4c00130)
Supplement: Supplementary file 1 — mg4c00130_si_001.pdf [file mg4c00130_si_001.pdf]

Supporting Information

Photocatalytic Papers Comprising Au@SnO_2 Nanocrystals Immobilized on Cellulose Nanofibers for Sustainable Dye Degradation

Yu-Chen Wei,[a] Huai-En Chang,[a] Pulikkutty Subramaniyan,[b] Shan-Chu Yu,[c] Yung-Jung Hsu,[a,d,e*] Tzu-En Lin[b*]

[a] Department of Materials Science and Engineering, National Yang Ming Chiao Tung University, Hsinchu 300093, Taiwan

[b] Institute of Applied Mechanics, National Taiwan University, Taipei 10617, Taiwan

[c] Institute of Biomedical Engineering, National Yang Ming Chiao Tung University, Hsinchu 300093, Taiwan

[d] Center for Emergent Functional Matter Science, National Yang Ming Chiao Tung University, Hsinchu 300093, Taiwan

[e] Institute of Integrated Research, Institute of Science Tokyo, Kanagawa 226-8503, Japan

*Email: yhsu@nycu.edu.tw (Yung-Jung Hsu); telin@iam.ntu.edu.tw (Tzu-En Lin)

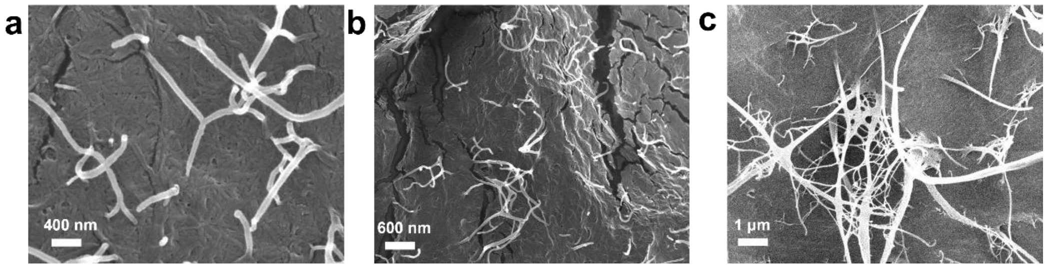

Figure S1. SEM images of pristine CNF at different magnifications.

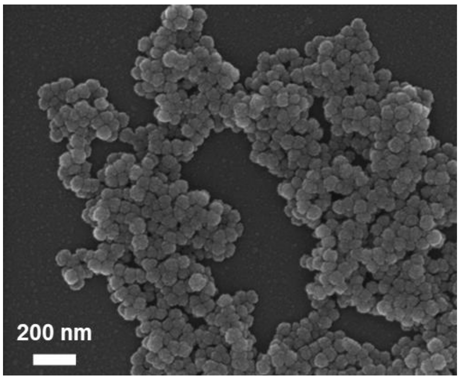

Figure S2. SEM image of individual Au@SnO_2.

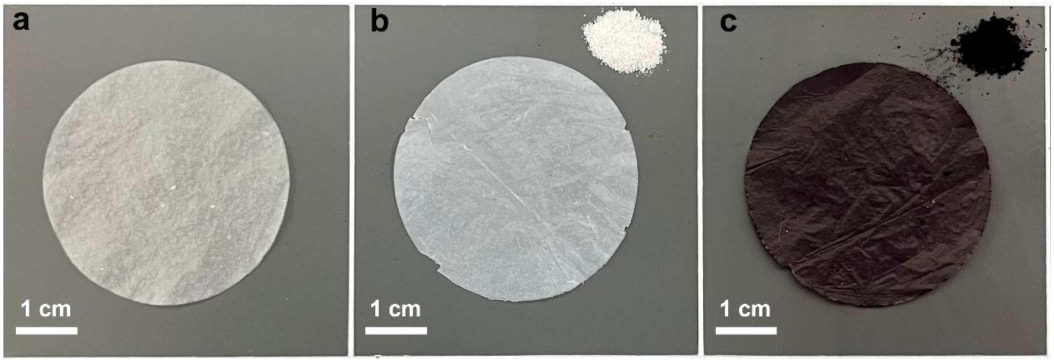

Figure S3. Photographs of (a) pristine CNF, (b) SnO_2 and SnO_2/CNF, (c) Au@SnO_2 and Au@SnO_2/CNF.

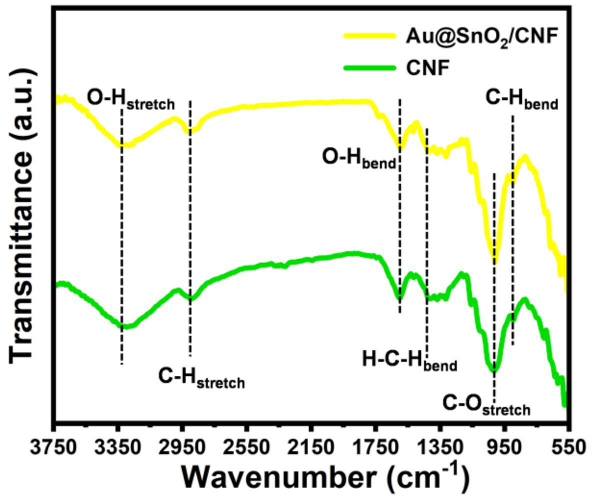

Figure S4. FTIR spectra for pristine CNF and Au@SnO$_2$/CNF.

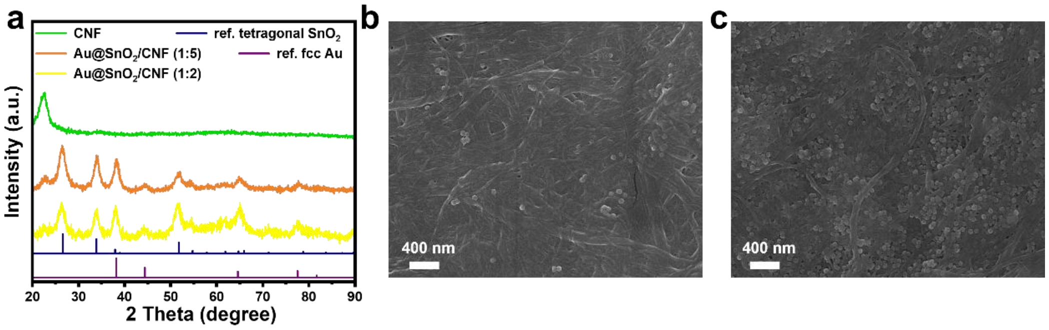

Figure S5. XRD patterns of pristine CNF and Au@SnO$_2$/CNF synthesized with Au@SnO2 to CNF ratios of 1:5 and 1:2. SEM images of (b) Au@SnO$_2$/CNF (1:5), (c) Au@SnO$_2$/CNF (1:2).

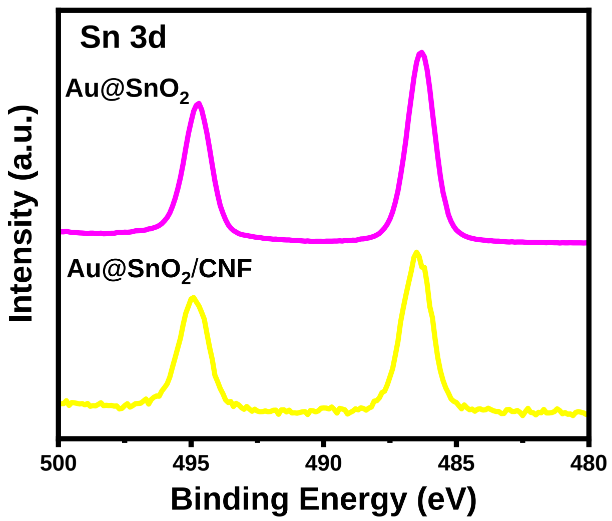

Figure S6. XPS Sn 3d spectra individual Au@SnO_2 and Au@SnO_2/CNF.

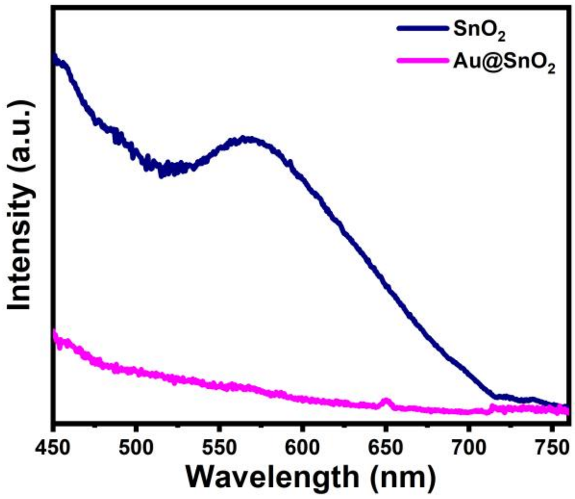

Figure S7. Steady-state PL spectra for individual Au@SnO_2 and pure SnO_2

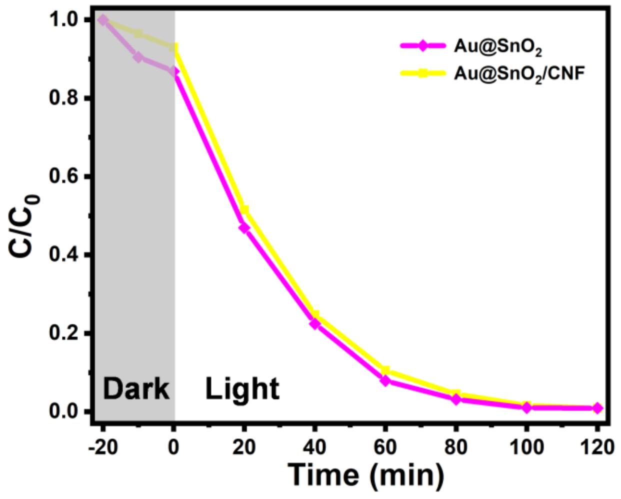

Figure S8. C/C_0 versus time plots for RhB degradation in the dark and under light illumination in the presence of individual Au@SnO_2 and Au@SnO_2/CNF.

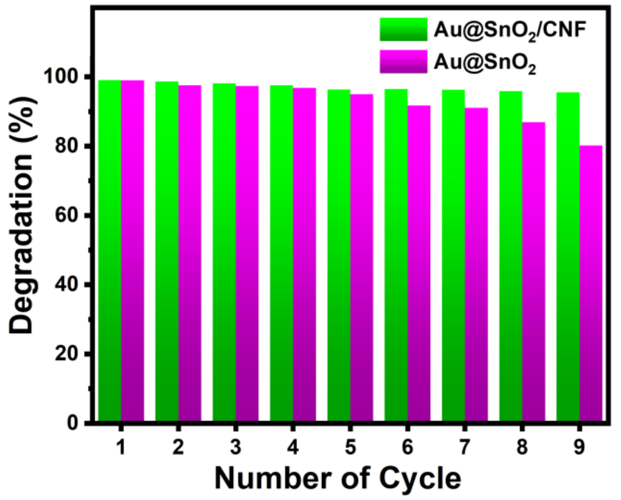

Figure S9. Comparison of RhB degradation performance between individual Au@SnO_2 and Au@SnO_2/CNF over multiple cycles.

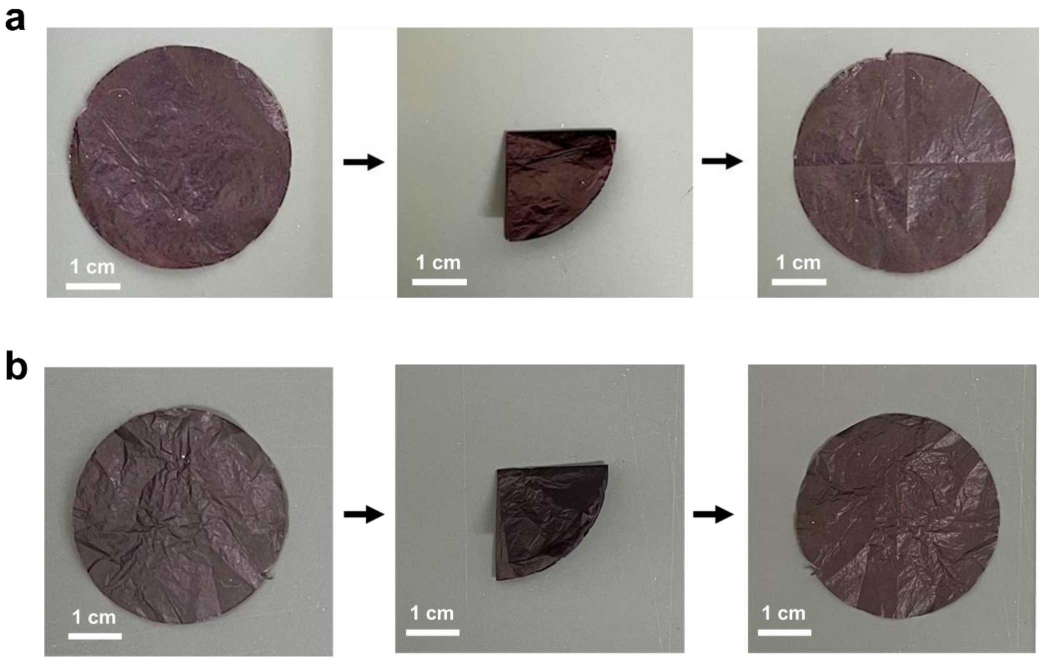

Figure S10. Photographs of Au@SnO_2/CNF NIP subjected to a folding test: (a) before and (b) after repeated use in RhB degradation.

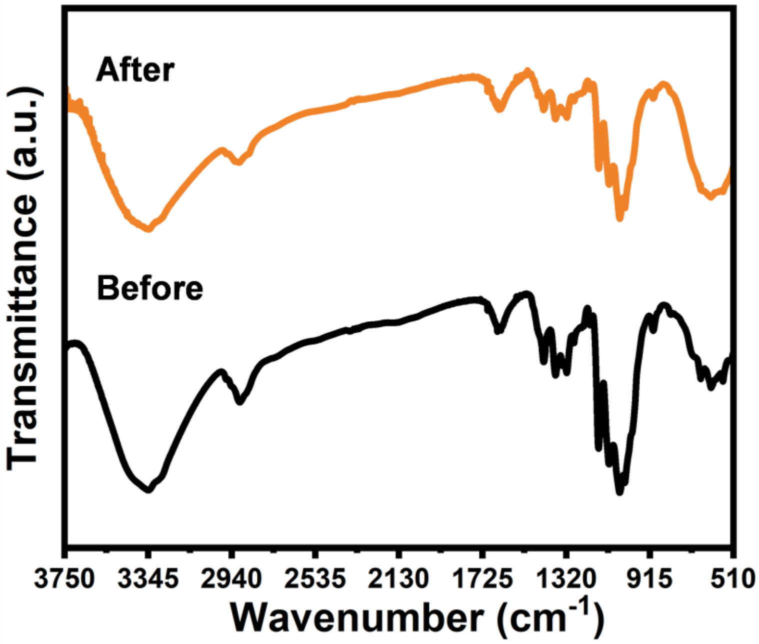

Figure S11. FTIR spectra of Au@SnO_2/CNF NIP before and after repeated use in RhB degradation.

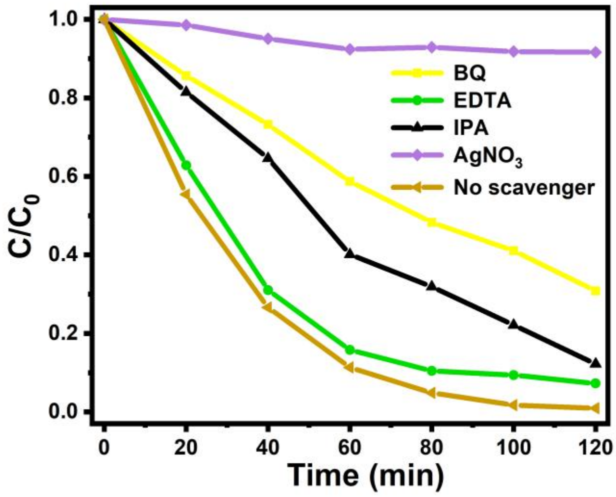

Figure S12. Results of scavenger experiments on Au@SnO_2/CNF NIP.
